# A novel referral system to optimize timing to specialized palliative care in patients with advanced cancer: an interventional study

**DOI:** 10.1093/oncolo/oyag428

**Published:** 2026-10-28

**Authors:** Giacomo Massa, Ernesto Zecca, Gabriele Tinè, Alessia Garavaglia, Antonino Tallarita, Andra D Dumitrascu, Eleonora Cristarella, Alessandra Pigni, Rosalba Miceli, Paolo G Casali, Lisa Licitra, Filippo G M deBraud, Cinzia Brunelli, Augusto F T Caraceni

**Affiliations:** Palliative Care, Pain Therapy and Rehabilitation Unit, Fondazione IRCCS Istituto Nazionale dei Tumori, via Giacomo Venezian n 1, Milano, 20133, Italy; Department of Clinical Sciences and Community Health, Dipartimento di eccellenza 2023–2027, Università degli Studi di Milano, Via della Commenda 19, Milano, 20122, Italy; Palliative Care, Pain Therapy and Rehabilitation Unit, Fondazione IRCCS Istituto Nazionale dei Tumori, via Giacomo Venezian n 1, Milano, 20133, Italy; Palliative Care, Pain Therapy and Rehabilitation Unit, Fondazione IRCCS Istituto Nazionale dei Tumori, via Giacomo Venezian n 1, Milano, 20133, Italy; Unit of Biostatistics for Clinical Research, Fondazione IRCCS Istituto Nazionale dei Tumori, Via Giacomo Venezian n1, Milano, 20133, Italy; Palliative Care, Pain Therapy and Rehabilitation Unit, Fondazione IRCCS Istituto Nazionale dei Tumori, via Giacomo Venezian n 1, Milano, 20133, Italy; Department of Medical Oncology, Fondazione IRCCS Istituto Nazionale dei Tumori, Via Giacomo Venezian n1, Milano, 20133, Italy; Department of Medical Oncology, Fondazione IRCCS Istituto Nazionale dei Tumori, Via Giacomo Venezian n1, Milano, 20133, Italy; Palliative Care, Pain Therapy and Rehabilitation Unit, Fondazione IRCCS Istituto Nazionale dei Tumori, via Giacomo Venezian n 1, Milano, 20133, Italy; Unit of Biostatistics for Clinical Research, Fondazione IRCCS Istituto Nazionale dei Tumori, Via Giacomo Venezian n1, Milano, 20133, Italy; Department of Medical Oncology, Fondazione IRCCS Istituto Nazionale dei Tumori, Via Giacomo Venezian n1, Milano, 20133, Italy; Department of Oncology and Hemato-Oncology, Università degli Studi di Milano, Via Santa Sofia 9, Milano, 20122, Italy; Department of Medical Oncology, Fondazione IRCCS Istituto Nazionale dei Tumori, Via Giacomo Venezian n1, Milano, 20133, Italy; Department of Oncology and Hemato-Oncology, Università degli Studi di Milano, Via Santa Sofia 9, Milano, 20122, Italy; Department of Medical Oncology, Fondazione IRCCS Istituto Nazionale dei Tumori, Via Giacomo Venezian n1, Milano, 20133, Italy; Department of Oncology and Hemato-Oncology, Università degli Studi di Milano, Via Santa Sofia 9, Milano, 20122, Italy; Palliative Care, Pain Therapy and Rehabilitation Unit, Fondazione IRCCS Istituto Nazionale dei Tumori, via Giacomo Venezian n 1, Milano, 20133, Italy; Palliative Care, Pain Therapy and Rehabilitation Unit, Fondazione IRCCS Istituto Nazionale dei Tumori, via Giacomo Venezian n 1, Milano, 20133, Italy; Department of Clinical Sciences and Community Health, Dipartimento di eccellenza 2023–2027, Università degli Studi di Milano, Via della Commenda 19, Milano, 20122, Italy

**Keywords:** early palliative care, referral tool, PROMs, healthcare resource

## Abstract

**Background:**

Early integration of specialist palliative care (PC) improves outcomes for patients with advanced cancer, yet implementation of needs-based referral remains inconsistent in routine oncology practice. The Palliative Care Referral System (PCRS) is a digital clinical decision-support tool designed to support referral using patient-reported symptoms, prognostic indicators, and clinical complexity. We evaluated the impact of PCRS implementation on referral patterns, patient-centered outcomes, healthcare utilization, and end-of-life (EoL) care.

**Patients and Methods:**

In this single-center prospective quasi-experimental pre–post implementation study, 380 patients with advanced solid tumors were enrolled before (pre-PCRS, *n* = 190) and after (post-PCRS, *n* = 190) PCRS implementation. During the post-PCRS phase, oncologists completed the PCRS at each outpatient visit. Patient and caregiver satisfaction, quality of life, healthcare utilization, and EoL indicators were assessed monthly for 6 months. Time to first PC referral was analyzed using competing-risk methods.

**Results:**

Overall 24-week referral rates were similar in the post-PCRS and pre-PCRS cohorts (21% vs 20%). However, referral timing and patterns changed across tumor types, with earlier referrals in breast and prostate cancer after PCRS implementation. The proportion of patients with at least 1 hospitalization was numerically lower after implementation (16% vs 27%; odds ratio, 0.53; 95% CI, 0.32-0.88; *P* = .017), but the difference was not significant after correction for multiple comparisons (*P* = .085). No significant differences were observed in patient or caregiver satisfaction, quality of life, emergency department visits, oncology visits, or PC visits. Indicators of aggressive EoL care were not significantly different between cohorts.

**Conclusions:**

PCRS implementation did not increase the overall referral rate but was associated with changes in referral timing and patterns across tumor types. Whether these changes reflect more appropriate, needs-based care and translate into lower healthcare utilization requires confirmation in larger studies.

Implication for practice Referral to specialist palliative care remains inconsistent despite guideline recommendations for early, needs-based integration. This study shows that implementing a workflow-integrated digital referral system can modify the timing and pattern of referral across tumor types, rather than the overall referral rate, while preserving oncologist judgment and without increasing outpatient resource utilization. Rather than replacing clinical decision-making, structured longitudinal assessment may help operationalize guideline recommendations within routine oncology practice and facilitate integration of specialist palliative care across diverse clinical settings.

## Introduction

In patients with advanced cancer, early referral to palliative care (EPC) has consistently demonstrated improvements in quality of life (QoL), psychological well-being, illness awareness, decision-making, and reductions in aggressiveness of end-of-life (EoL) care.^1–3^ EPC also confers benefits for caregivers and optimizes healthcare resource utilization.^4^ These positive effects have been confirmed across multiple contexts, including solid and hematologic malignancies.^1^^,^^5–8^ Furthermore, several EPC interventions are well accepted by both patients and caregivers, particularly when accompanied by a clear perception of effectiveness.^9–11^

Since the landmark randomized trial by Temel et al.^12^ in 2010, several studies have investigated EPC using heterogeneous patient populations (various malignancies, clinical settings), screening strategies (sequential enrollment vs. symptom- or criteria-based selection), types of palliative care intervention (physician-led consultations, nurse-led visits, telephone-based assessments), and measured outcomes.^1^ Despite this heterogeneity, the 2017 American Society of Clinical Oncology (ASCO) guidelines recommended referral to palliative care within 8 weeks of a diagnosis of advanced cancer.^13^ However, it is necessary to consider the complexity of different cancer types and treatments, the multidimensional needs of patients and caregivers, and the variability of healthcare system contexts with limited specialized palliative care resources. On the other side, to advance EPC, it is essential to address the multiple barriers regarding integration between specialized palliative care and medical oncology services.^14^ To date, only 8% of advanced cancer patients in the United States receive EPC within 8 weeks of diagnosis of metastatic disease.^15^ At the same time, disparities in access are even more pronounced in low- and middle-income countries.^16^

Moreover, implementing personalized palliative care referral is critical to ensuring the optimal allocation of healthcare resources. Proper integration not only reduces EoL care aggressiveness and unnecessary hospitalizations or emergency room visits but also helps address the challenges posed by the global shortage of healthcare personnel. The most recent ASCO guidelines on integrating oncology and palliative care highlight that referral decisions should consider disease aggressiveness, prognostic indicators, patient-reported symptom burden, and unmet needs.^17^ However, despite these recommendations, validated criteria or tools for guiding EPC referral are lacking. Several studies have based referral on time from advanced disease diagnosis, others on symptom burden.^1^^,^^18–21^ In addition, expert consensus initiatives have proposed referral criteria, including major and minor indicators for EPC eligibility.^22^^,^^23^ However, decisions largely remain at the discretion of individual oncologists, with no practical tools validated for routine clinical use.

To address this gap, we developed the Palliative Care Referral System (PCRS), a digital, rapid-to-complete scoring tool co-designed with oncologists and other professionals to guide in identifying patients who may benefit from EPC.^24^^,^^25^

The present study aims to evaluate the effect of implementing the PCRS in outpatient oncology clinics on time to palliative care referral, patient satisfaction with care and QoL, caregivers’ satisfaction with care, healthcare resource utilization, and EoL care aggressiveness criteria.

## Methods

### Study design

A quasi-experimental, longitudinal, pretest-posttest study was carried out at the Fondazione IRCCS Istituto Nazionale dei Tumori di Milano. Two independent cohorts from 6 outpatient cancer clinics (breast, thoracic, gastrointestinal, genitourinary, head and neck, and sarcoma) were sequentially enrolled before (pre-PCRS cohort) and after (post-PCRS cohort) the PCRS implementation.

### Setting

The study was conducted at a comprehensive cancer center with a dedicated Palliative Care Unit-Hospice, Pain Therapy and Rehabilitation, providing outpatient PC 5 days/week (3 physicians, 4 nurses), inpatient consultation, and links to home care and hospice. Before PCRS implementation, specialist PC referral was based on individual oncologist judgment. Primary palliative care, including symptom management and diagnosis/prognosis communication, was provided by oncology teams; psychological support was available through a dedicated psycho-oncology service PC. Referral was mainly driven by diagnosis and perceived proximity to end of life rather than validated needs-based criteria. The PCRS was designed during the codesign phase to address these barriers by routinely making symptom burden, prognostic indicators, and clinical complexity visible at the point of care.^25^

### Target population

Adult patients with a diagnosis of inoperable locally advanced and/or metastatic cancer not eligible for curative treatment were eligible for the study. Patients already receiving palliative care or with cognitive impairments that could interfere with study participation were excluded (details can be found in the protocol paper).^24^ Written informed consent was obtained before enrollment from all patients and participating caregivers using the same form and procedure in both phases.

### Intervention: the PCRS

The PCRS was designed to identify patients with advanced cancer and palliative care needs to be referred to the palliative care outpatient clinic. To facilitate implementation, oncologists working in the clinics were involved in the codesign and modes of implementation of the tool.^25^ The PCRS includes 14 items across 3 domains (Table S1): patient-reported outcome measures (PROMs, 3 items), performance status, performance prognostic indicators and patient preferences (ProPlus, 4 items), and clinical complexity (ClinC, 7 items).^25^ A digital version of the PCRS was integrated into the electronic medical record (EMR) of the outpatient oncology clinic to ensure easy accessibility for clinicians during visits (Figure S2, see online supplementary material for a color version of this figure). To further facilitate the PCRS implementation in routine outpatient cancer care, before the visit, a nurse administered the PROMs included in the PCRS’ symptoms/needs-domain (Edmonton Symptom Assessment Scale for symptoms and part of the Distress Thermometer for psycho-social issues).^26^^,^^27^ The results of such assessment were entered in the PRCS digital form within the EMR, validated, and completed by the oncologist during the visit. The PCRS did not produce a referral recommendation based on a predefined threshold, and no guidance was given as to which item combinations should warrant referral; the form was displayed within the EMR, and the decision to refer remained at the oncologist’s discretion.

The PCRS form was filled out at each oncology visit, with a minimum time interval of 1 week, until the patient completed the 6-month follow-up period or was referred to the Palliative Care (PC) unit.

#### Outcome measures and data collection

The primary outcome was the patient’s satisfaction with care, measured by the FAMCARE P13.^28^^,^^29^ Secondary outcomes were patient’s QoL (EORTC-QLQ-C15), caregiver’s care satisfaction (FAMCARE),^28–30^ use of healthcare resources during the 6-month study follow-up (outpatient or residential PC referral, time to PC referral, number of PC and oncology visits, number of hospitalization and emergency room visits) and EoL care aggressiveness indicators during the last month of life for patients who died within the study follow-up period. All outcomes but caregivers’ satisfaction were assessed monthly for a duration of at most 6 months; caregivers’ satisfaction was assessed at baseline, at month 3, and at month 6.^30^^,^^31^ More details of outcome assessment are reported in the protocol paper.^24^ FAMCARE-P13 comprises 13 items on a 5-point Likert scale (1 = very dissatisfied, 5 = very satisfied), higher scores indicate greater satisfaction. The 20-item caregiver FAMCARE is scored similarly. The EORTC QLQ-C15-PAL global health status/quality of life scale comprises 2 seven-point items transformed to 0-100; higher scores indicate better quality of life, while symptom scales use the same transformation, with higher scores indicating greater symptom burden. Diagnoses prompting hospitalization or emergency room (ER) attendance were not systematically coded. Hospitalizations and ER attendances were recorded separately; ER attendance leading to admission was counted as both.

#### Sample size

Power considerations carried out in the study planning phase indicated that 150 patients in each group (pre-PCRS and post-PCRS cohorts) would allow a power of 0.9 to detect a standardized effect size of 0.375. This effect size was defined as the between-group difference in mean change from baseline to the average of the repeated follow-up FAMCARE-P13 scores, divided by the common standard deviation (alpha error = 0.05). The sample size was increased by 30 patients per group during the final phase of the study. This was done to include patients with breast cancer, ensuring a broader and more representative spectrum of the outpatient cancer patients, also in view of the predefined interest in conducting cancer-specific exploratory subgroup analyses. Ten additional patients were included in each group to account for those who were enrolled but failed to complete at least 1 assessment of the main outcome measure. In total, 190 patients per group were enrolled, which allows a power of 90% to detect an effect size of 0.332. Furthermore, different cancer types may exhibit distinct patterns of PC referral and variable effects on care experience. Thus, cancer type was defined as a clinically relevant stratification variable for exploratory subgroup analyses to describe how the PCRS performs across distinct tumor-specific clinical contexts. Consequently, the enlargement of the sample size also improved the study’s ability to explore tumor-specific subgroup analyses.

### Statistical analysis

#### Main analyses

Continuous variables were reported as medians with interquartile ranges (IQRs), and categorical variables as absolute and relative frequencies.

Given the non-randomized study design, comparisons between the 2 intervention groups were adjusted for potential confounders. A logistic regression model was fitted with the intervention group as the dependent variable and age, Eastern Cooperative Oncology Group Performance Status (ECOG-PS), tumor type, number of metastatic sites, ongoing therapy, and time elapsed between the diagnosis of advanced/metastatic disease and study entry as covariates. This model was used to estimate the propensity score for receiving the intervention and to derive the corresponding inverse probability of treatment weights (IPTW).^32^^,^^33^ These weights were subsequently incorporated into comparative analyses.

According to the study protocol^24^ an independent samples t-test was employed to compare the between-group difference in the mean change from baseline to the average of repeated follow-up FAMCARE-P13 scores (main outcome); for sensitivity reasons, between-group comparison was repeated using a weighted 2-sample Welch’s *t*-test, with group-specific means and variances estimated by weighting each observation according to its IPTW.

Analogous adjusted analyses were conducted for secondary patient-reported outcomes including patient QoL and caregiver satisfaction, using EORTC-QLQ-C15 and FAMCARE scores respectively.

For the secondary outcome of PC referral, the endpoint was time to first palliative care referral (TFPCR), defined as the interval from study entry to the first palliative care referral (FPCR). Death occurring before PC activation was treated as a competing event, while patients alive without PC activation at the end of the 6-month follow-up were censored at their last assessment.

The cumulative incidence of FPCR in the 2 study groups (post-PCRS vs pre-PCRS) was estimated using the IPTW-weighted Aalen-Johansen estimator.^34^ The area under the cumulative incidence curves was quantified using the IPTW-weighted restricted mean time lost (RMTL). The between-group difference in RMTL represents the average time to FPCR lost (or gained) following the introduction of PCRS.^35^ Both cumulative incidence and RMTL were evaluated at 4, 8, 12, and 24 weeks.

Further comparisons were performed using IPTW-adjusted Fine and Gray models to compare the 2 groups in terms of sub-distribution hazard ratios (sHRs) and their corresponding 95% CIs. Healthcare resource utilization and aggressiveness of EoL care were summarized descriptively. IPTW-adjusted logistic regression models were estimated to compare the 2 groups in terms of hospitalization occurrence, access to E.R., and systemic oncologic therapy in the last 30 days of life. Odds ratios (ORs) along with 95% CI were reported only when model convergence was achieved. All these models were therefore adjusted for potential clinical confounders through IPTW, using the propensity score model described above (age, ECOG-PS, tumor type, number of metastatic sites, ongoing therapy, and time from advanced/metastatic diagnosis to study entry). This adjustment for confounding is distinct from the correction for multiple comparisons, which does not modify the effect estimates but controls the false discovery rate across the set of tests performed. To account for multiplicity, *P*-values for the 5 healthcare utilization and end-of-life comparisons were adjusted using the Benjamini–Hochberg false discovery rate procedure (both unadjusted and adjusted *P*-values are reported).

#### Subgroup analyses

The increased sample size enabled tumor-specific subgroup exploratory analyses, as patterns of PC referral may vary across different tumor types.

Analogous to the main analyses, IPTW were estimated to account for potential confounding, with propensity scores derived from the logistic regression model described in the main analyses, excluding tumor type from the set of covariates.

The cumulative incidence of FPCR in the 2 study groups was estimated by stratifying by tumor type using the IPTW-weighted Aalen–Johansen estimator, and IPTW-adjusted RMTL estimates were also reported.

Moreover, Fine and Gray models, adjusted using IPTW, were fitted to compare the 2 groups in subsets defined by tumor type, reporting each sHR and their corresponding 95% CIs.

An additional exploratory analysis was performed by grouping tumor types into 3 prognostic categories: poor prognosis (biliary-pancreatic-gastric cancers, thoracic-other tumors, and bladder and kidney cancers), intermediate prognosis (sarcomas, lung, head and neck, and colorectal cancers), and favorable prognosis (breast and prostate cancers). This approach was used to limit the number of strata and to improve the precision and stability of subgroup estimates compared with modeling each tumor type separately. To formally assess whether the effect of PCRS implementation on TFPCR differed across prognostic groups, IPTW-adjusted Fine and Gray models were fitted within each subgroup, and category-specific sHRs with their corresponding 95% Cis were reported. IPTW for this analysis were estimated using propensity scores derived with the logistic regression model described in the main analyses, using the prognosis category as covariate in place of tumor type.

All tests, for both main and subgroup analyses, were 2-sided, with *P*-values <.05 considered statistically significant. All statistical analyses were performed using RStudio (version 4.4.1).

## Results

A total of 380 patients (pre-PCRS, *n* = 190; post-PCRS, *n* = 190) were enrolled across pre-specified tumor types (Figure 1) from January 13, 2021 to March 22, 2024. Three hundred and seventy-two (98%) had ≥2 assessments, and 6-month follow-up was reached by 157 (83%) in pre-PCRS and 169 (89%) in post-PCRS (Table S1). Median age was 66 years (IQR, 57-73), and 46% were women. Some baseline imbalances were present: ECOG PS (0 in 50% of pre-PCRS group vs 63% in post-PCRS; *P* = .03), time interval from advanced/metastatic diagnosis to enrollment (median, 4.05 in pre-PCRS vs 8.4 months in post-PCRS *P* = .02), and proportion of patients on systemic therapy (86% in pre-PCRS vs 98% in post-PCRS; *P* < .001) (Table 1). Eligible patients who were not enrolled are detailed in Figure 1. During the post-PCRS phase, the PCRS form was completed in 83% of the eligible oncology visits over the first 6 months.

**Figure 1.**
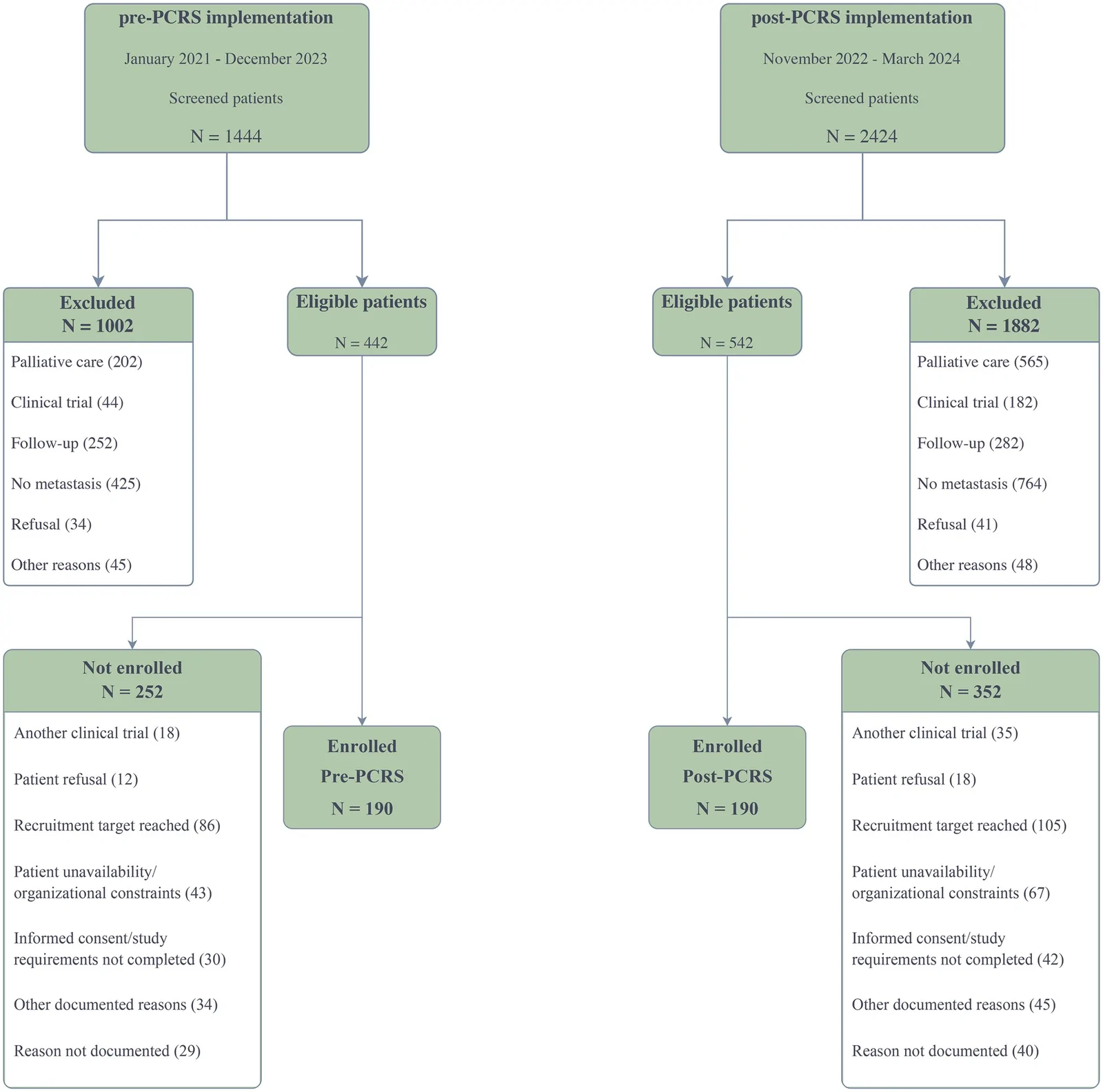
Study flow diagram.

**Table 1.** Patients’ demographic and clinical characteristics.

Characteristics	Overall, *N* = 380^a^	Pre-PCRS, *N* = 190	Post-PCRS, *N* = 190	*P*-value^b^
**Age**	66.1 (56.8, 73.2)	64.8 (56.1, 72.6)	67 (56.9, 73.3)	.31
**Gender**				.76
** Female**	173 (45.5%)	88 (46.3%)	85 (44.7%)	
** Male**	207 (54.5%)	102 (53.7%)	105 (55.3%)	
**Type of tumor**				.96
** Lung**	59 (15.5%)	32 (16.8%)	27 (14.2%)	
** Thoracic/other**	9 (2.4%)	4 (2.1%)	5 (2.6%)	
** Colorectal**	22 (5.8%)	8 (4.2%)	14 (7.4%)	
** Biliary pancreatic gastric**	39 (10.3%)	21 (11.1%)	18 (9.5%)	
** Head and neck**	65 (17.1%)	33 (17.4%)	32 (16.8%)	
** Sarcomas**	62 (16.3%)	30 (15.8%)	32 (16.8%)	
** Breast**	61 (16.1%)	31 (16.3%)	30 (15.8%)	
** Prostate**	18 (4.7%)	9 (4.7%)	9 (4.7%)	
** Bladder and kidney**	45 (11.8%)	22 (11.6%)	23 (12.1%)	
**No. of metastatic sites**				.08
** 0**	33 (8.7%)	11 (5.8%)	22 (11.6%)	
** 1**	135 (35.5%)	76 (40.0%)	59 (31.1%)	
** 2**	117 (30.8%)	58 (30.5%)	59 (31.1%)	
** 3**	57 (15.0%)	28 (14.7%)	29 (15.3%)	
** 4**	30 (7.9%)	11 (5.8%)	19 (10.0%)	
** 5**	6 (1.6%)	5 (2.6%)	1 (0.5%)	
** 6**	2 (0.5%)	1 (0.5%)	1 (0.5%)	
**ECOG PS**				.03
** 0**	215 (56.6%)	95 (50.0%)	120 (63.2%)	
** 1**	152 (40.0%)	88 (46.3%)	64 (33.7%)	
** 2**	13 (3.4%)	7 (3.7%)	6 (3.2%)	
**Time elapsed from advanced/metastatic diagnosis and baseline (months)**	6.91 (0.0, 37.1)	4.05 (0.0, 29.3)	8.4 (0.2, 45.1)	.02
** Missing**	2	1	1	
**Ongoing therapy (<1 month)**	351 (92.4%)	164 (86.3%)	187 (98.4%)	<.001
**Educational qualification**				.82
** Primary school**	105 (27.8%)	55 (29.3%)	50 (26.3%)	
** Secondary school**	187 (49.5%)	92 (48.9%)	95 (50.0%)	
** Bachelor’s degree**	6 (1.6%)	2 (1.1%)	4 (2.1%)	
** Master’s degree**	80 (21.2%)	39 (20.7%)	41 (21.6%)	
** Missing**	2	2	0	
**Presence of a caregiver**	299 (78.7%)	146 (76.8%)	153 (80.5%)	.38

Abbreviation: PCRS, Palliative Care Referral System.

a Data are median (IQR) or *n* (%).

b *P*-values compare the pre-PCRS group and the post-PCRS using the Wilcoxon Mann-Whitney test or the Fisher exact test, Fisher Freeman Halton Test.

### Patient and caregiver satisfaction with care and patient QoL

For the primary endpoint, the between-group difference in mean change from baseline to the average of repeated follow-up FAMCARE-P13 scores was not statistically significant (between-group difference, 0.03; 95% CI, −0.05 to 0.11; *P* = .495). Similarly, differences in QoL (2.56; 95% CI, −2.25 to 7.37; *P* = .295) and caregiver satisfaction (0.05; 95% CI, −0.04 − 0.16; *P* = .254) were small and not statistically significant. IPTW-adjusted analyses yielded similar results (Table 2).

**Table 2.** Differences between the 2 groups for FAMCARE-P13 (main study outcome), FAMCARE, and EORTC QLQ-C15-PAL questionnaire total scores.

	Pre-PCRS	Post-PCRS		
	Mean (95% CI)	Mean (95% CI)	Between-group difference (95% CI)	*t*-test *P*-value
**FAMCARE P13**				
	*N = 1*80	*N = 1*84		
** Baseline score**	4.4 (4.3-4.4)	4.4 (4.3-4.4)		*N =* 180
** Average follow-up scores**	4.3 (4.3-4.4)	4.3 (4.3-4.4)	0.03 (−0.05 to 0.11)	.495
**FAMCARE**				
	*N =* 129	*N =* 143		
** Baseline score**	4.3 (4.2-4.4)	4.3 (4.3-4.4)		*N =* 129
** Average follow-up scores**	4.3 (4.2-4.4)	4.3 (4.2-4.4)	0.05 (−0.04 to 0.16)	.254
**EORTC QLQ-C15 PAL**				
	*N =* 180	*N =* 184		
** Baseline score**	72 (69-75)	74 (71-77)		*N =* 180
** Average follow-up scores**	71 (69-73)	70 (68-73)	2.56 (−2.25 to 7.37)	.295

The average follow-up score was calculated as the mean represents the average of the questionnaire scores collected at the prespecified timepoint. FAMCARE-P13 comprises 13 items on a 5-point Likert scale; higher scores indicate greater satisfaction. The 20-item caregiver FAMCARE is scored similarly. The EORTC QLQ-C15-PAL global health status/quality of life scale comprises 2 seven-point items transformed to 0-100; higher scores indicate better quality of life.

Abbreviation: PCRS, Palliative Care Referral System.

### Palliative care referral

All the patients referred to specialist PC in the pre-PCRS and in the post-PCRS cohort attended at least 1 PC assessment, at a median of 7 days from referral. Over the 6-month follow-up, 183 and 124 PC visits were recorded in the pre-PCRS and post-PCRS cohorts, respectively. Among patients attending the PC clinic, the median number of PC visits after referral was 5 (Q1-Q3, 2-8) and 4 (Q1-Q3, 2-6), respectively. The total number of oncology visits was comparable in the 2 cohorts (1231 vs 1196; median 6 [Q1-Q3, 4-9] vs 6 [Q1-Q3, 4-8]), as was the median number of oncology visits after PC referral (2 [Q1-Q3, 0.75-7] vs 3 [Q1-Q3, 2-6]), indicating that specialist PC involvement was additive to, and did not replace, oncological follow-up. The cumulative incidence of FPCR was similar between the 2 study groups at 4, 8, 12, and 24 weeks (24 weeks referral rate 20% pre-PCRS vs 21% post-PCRS, *P* = .89; Figure 2; Table S2). Indeed, RMTL differences were small and non-significant at all time points (24 weeks: ΔRMTL= +0.2 weeks, *P* = .81; Table S3). The Fine-Gray competing risks model yielded consistent estimates for the chance of PC referral (sHR : 0.96, 95% CI, 0.6-1.5; *P* = .85).

**Figure 2.**
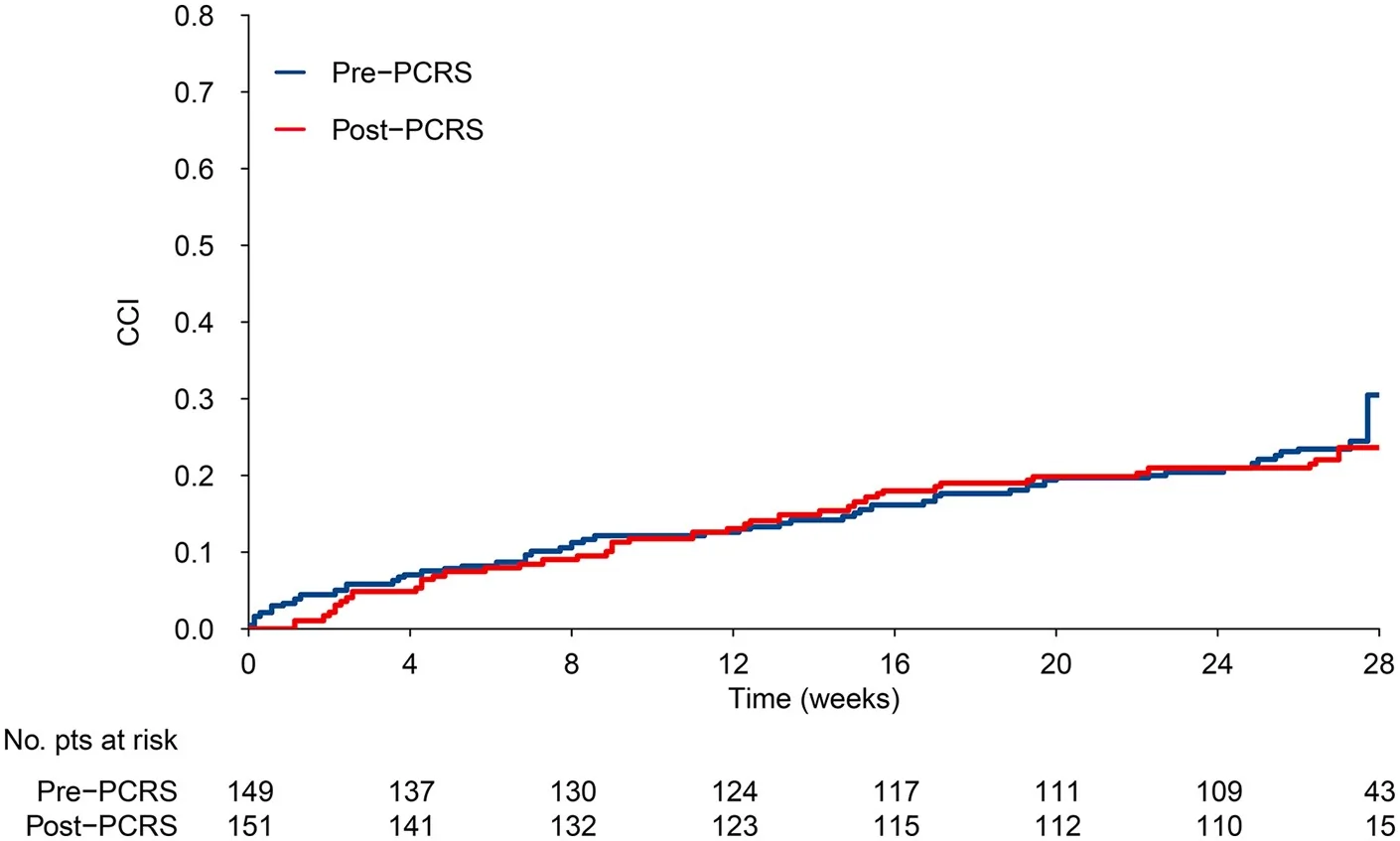
First palliative care referral cumulative incidence curves stratified by group (pre-PCRS vs post-PCRS). IPTW-adjusted crude cumulative incidence curves of FPCR for the pre-PCRS and post-PCRS groups are largely overlapping. Abbreviations: CCI, crude cumulative incidence; FPCR, first palliative care referral; IPTW, inverse probability of treatment weighting; PCRS, Palliative Care Referral System; pts, patients.

However, in tumor-specific subgroup analyses, patterns of FPCR differed between study groups. In prostate cancer, the post-PCRS group showed a higher cumulative incidence across time points (24 weeks: 0% pre-PCRS vs 70% post-PCRS [95% CI, 39%-100%]; *P* = .002; Figure 3H; Table S4). In biliary-pancreatic-gastric cancers, the cumulative incidence of FPCR was lower after PCRS implementation (24 weeks : 47% [95% CI, 25%-70%] pre-PCRS vs 33% [95% CI, 9%-56%] post-PCRS; *P* = .05; Figure 3D). In head and neck tumors, the FPCR showed a lower incidence at each time point after PCRS implementation (Figure 3E).

**Figure 3.**
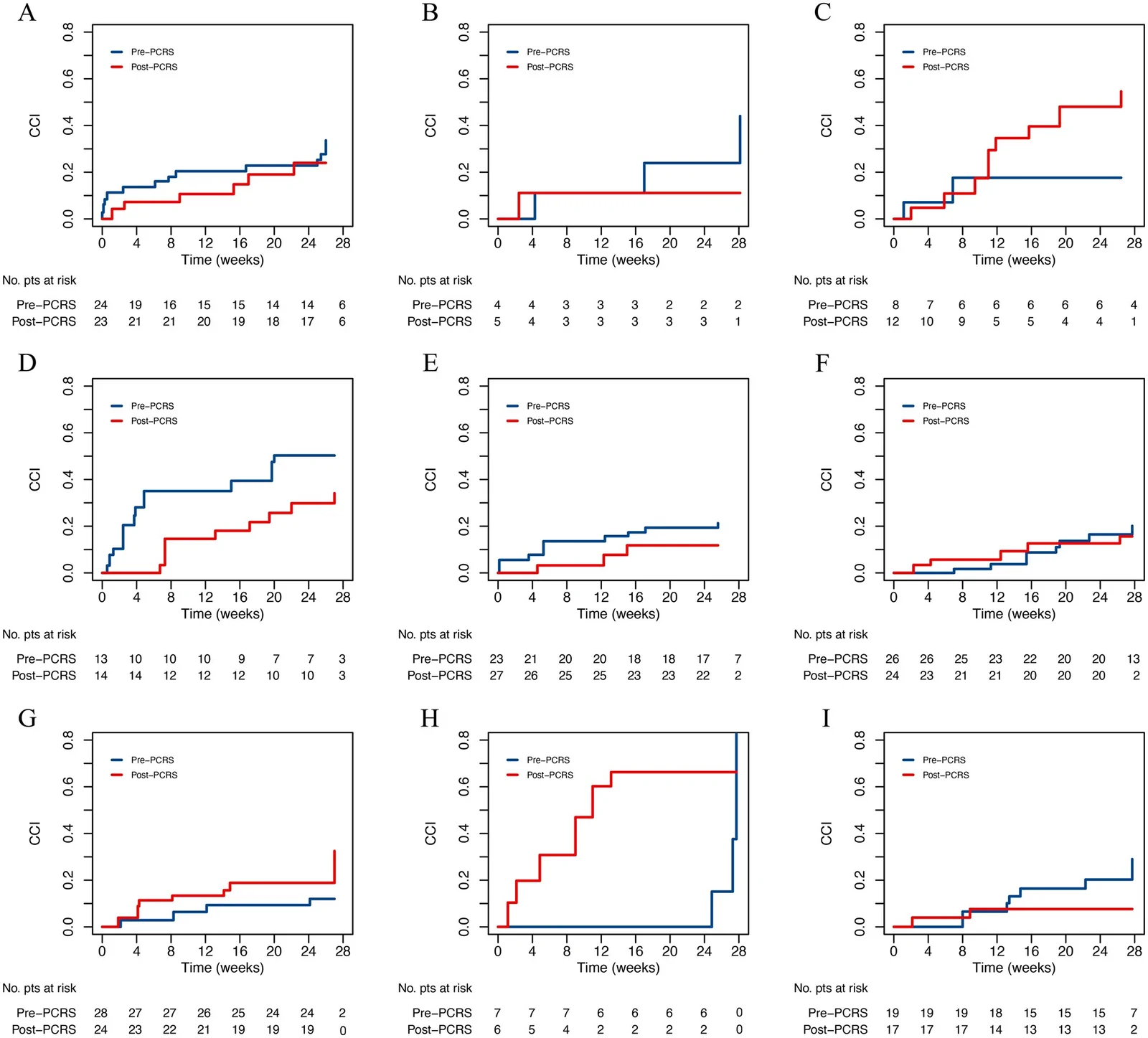
First palliative care referral cumulative incidence curves stratified by group (pre-PCRS vs post-PCRS) by each tumor type. The panels displayed the IPTW-adjusted crude cumulative incidence curve of FPCR for lung (A), thoracic other (B), colorectal (C), biliary/pancreatic/gastric (D), head and neck (E), sarcomas (F), breast (G), prostate (H), and bladder and kidney (I) cancers. For each tumor type, pre-PCRS curves are depicted in blue and post-PCRS in red. Abbreviations: CCI, crude cumulative incidence; FPCR, first palliative care referral; IPTW, inverse probability of treatment weighting; PCRS, Palliative Care Referral System; pts, patients.

When cancers were aggregated by prognosis, the favorable-prognosis group (breast-prostate) showed a higher FPCR cumulative incidence after PCRS implementation (24 weeks: pre-PCRS 6% [95% CI, 0%-14%] vs 30% [95% CI, 16%-45%] post-PCRS; *P* = .05). The poor-prognosis group (biliary-thoracic/other-bladder and kidney), although not statistically significant, consistently exhibited lower FPCR cumulative incidence at each time point (Figure S1 [see online supplementary material for a color version of this figure] and Table S5). The intermediate-prognosis group referral (sarcomas-lung-head and neck-colorectal) was similar between groups across all time points (24 weeks: pre-PCRS 21% [95% CI, 13%-30%] vs 19% [95% CI, 12%-27%] post-PCRS; *P* = .54). Patients referred to specialist PC had higher PCRS scores than non-referred patients: on the 0-24.5 composite scale, the median total score was 2 among patients who were not referred and 5 at the time of referral among those referred.

The RMTL, which summarizes the accrued FPCR over time, was higher in the post-PCRS group for breast–prostate cancer. Specifically, at 12 and 24 weeks, post-PCRS patients were referred approximately 1.3 and 4.1 weeks earlier than those in the pre-PCRS group (*P* = .032 and *P* = .008, respectively; Table S6).

The IPTW-adjusted Fine and Gray models yielded a sHR of 2.25 (95% CI, 0.95-5.34, *P* = .065) for the favorable-prognosis tumors, 0.84 (95% CI, 0.47-1.50, *P* = .560) for intermediate-prognosis tumors and 0.68 (95% CI, 0.31-1.51, *P* = .347) for the poor-prognosis tumors (Table S7).

### Healthcare utilization and end-of-life indicators

Over 6 months, the proportion of patients with at least 1 hospitalization was higher in the pre-PCRS group than in the post-PCRS group (27% vs 16%), with an IPTW-adjusted OR (post-PCRS vs pre-PCRS) of 0.53 (95% CI, 0.32-0.88; *P* = .017). ER visits at any time during follow-up were slightly lower after PCRS implementation (16% pre- vs 11% post-PCRS; OR, 0.66; 95% CI, 0.36-1.21; *P* = .224; Table 3). After adjustment for multiple comparisons (Benjamini–Hochberg procedure), the difference in hospitalizations was no longer statistically significant (adjusted *P* = .085). Deaths were more frequent in the pre-PCRS group (14%) than in the post-PCRS group (6%). Regarding EoL indicators, patients were more frequently hospitalized in the pre-PCRS group (44%) than in the post-PCRS group (25%) (OR, 0.42; 95% CI, 0.09-1.89; *P* = .301). Emergency room visits at EoL were also more frequent in the pre-PCRS group compared with the post-PCRS group (37 vs 17% and OR 0.34; 95% CI, 0.06-1.87; *P* = .280). Systemic oncologic therapy in the last 30 days of life was received by 30% and 33% of patients in the pre-PCRS and post-PCRS groups, respectively (OR, 1.19; 95% CI, 0.28-5.10; *P* = .991). However, none of these differences reached statistical significance and the number of events is low (Table 3).

**Table 3.** Healthcare resource utilization and events during the study period and in the end-of-life.

	Pre-PCRS	Post-PCRS	OR (95% CI)	*P*-value
	(*N* = 190)	(*N* = 190)		
** Hospitalization, *N* (%)**	51 (27)	31 (16)	0.53 (0.32-0.88)	.017
** ER visit, *N* (%)**	30 (16)	21 (11)	0.66 (0.36-1.21)	.224
**Dead patients**				
** Deceased, *N* (%)**	27 (14)	12 (6)	-	-
**Events in the 30 days prior to death**				
** Hospitalization, *N* (%)**	12 (44)	3 (25)	0.42 (0.09-1.89)	.301
** ER visit, *N* (%)**	10 (37)	2 (17)	0.34 (0.06-1.87)	.280
** Systemic oncologic therapy, *N* (%)**	8 (30)	4 (33)	1.19 (0.28-5.10)	.991

OR were estimated using IPTW-adjusted logistic regression. *P*-values are not adjusted for multiple comparisons; Benjamini-Hochberg adjusted *P*-values were .085 for hospitalization, .376 for ER visits, .376 for hospitalization, and .376 for ER visits in the last 30 days of life, and .991 for systemic oncologic therapy in the last 30 days of life.

Abbreviations: ER, emergency room visits; IPTW, inverse probability of treatment weighting; OR, odds ratio; PCRS, Palliative Care Referral System.

## Discussion

Identification of cancer patients who may benefit from early outpatient palliative care remains a research priority in order to optimize the well-demonstrated benefits of EPC integration in oncology.^1^^,^^12^^,^^36^ Overall, evidence supports personalized PC referral as a strategy to improve QoL and EoL care.

This study attempted to define clinically meaningful criteria for referring cancer outpatients to a PC service. The PCRS was designed based on literature suggestions, clinical experience, and by consensus within a multidisciplinary codesign procedure.^24^^,^^25^ The study showed no significant differences between the pre-PCRS and post-PCRS implementation cohorts in patient satisfaction with care, overall QoL, or caregiver satisfaction. Similar findings have been reported by other authors when comparing different referral-triggering systems for initiating palliative care (eg, STEP trial by Zimmermann et al.).^20^ Likewise, the overall number of palliative care referrals and their timing did not show a significant global change in the post-PCRS period compared with the pre-PCRS cohort (Figure 2).

However, a more nuanced pattern emerged. Although the global interaction *P*-value at multivariable analysis was not statistically significant, when stratifying by tumor type, referral timing appeared to be modified by the use of the PCRS: in tumor with an expected poor prognosis, referral showed a propensity toward postponement in the post-PCRS cohort, whereas patients with cancers associated with an expected good prognosis were referred earlier after the PCRS implementation (Figure 3).

This may reflect the influence of underlying malignancy on referral decisions before PCRS implementation. PCRS may help clinicians consider clinical indicators (objective signs domain) and patient-reported concerns, drawing attention to otherwise overlooked red flags. Thus, patients with more indolent diseases and higher PCRS scores were referred earlier than those with aggressive tumors but no additional PCRS-identified concerns. Referral may therefore have been more disease-driven before PCRS and more influenced by individual patient needs thereafter.

Overall, the main contribution of PCRS implementation appears to be a change in the timing and pattern of referral across tumor types rather than an increase in the overall referral rate, which was similar in the 2 cohorts. The lower proportion of hospitalized patients observed after implementation did not remain statistically significant after correction for multiple comparisons, and EoL indicators did not differ significantly. These findings should therefore be considered hypothesis-generating and do not demonstrate an effect of the PCRS on healthcare utilization.

Earlier referral, however, is not per se a marker of better care. A recent stepped-care trial^37^ showed that a needs-triggered, model was non-inferior to monthly early palliative care for quality of life while requiring fewer specialist visits, indicating that the referral precision may be more relevant than early referral. The clinical value of the observed redistribution of referrals cannot therefore be inferred from timing alone. In patients with indolent disease, low symptom burden, and needs adequately addressed by primary palliative care within the oncology team, earlier specialist referral may increase visits and treatment burden without proportional benefit; conversely, a later referral in patients with aggressive disease is appropriate only if needs remain low over time. Since no external reference standard of need-concordant referral was applied, our data do not establish whether referrals made after PCRS implementation were more appropriate, and future studies should link referral timing to patient-level needs and outcomes.

At our center, routine collaboration between oncology services and the palliative care clinic is longstanding,^38^ although not previously standardized using defined criteria. Symptom control and psychological support are also available through oncology and psycho-oncology services. This may have produced a ceiling effect for quality of life and overall PC referral, reducing study sensitivity. In fact, 97% had an ECOG PS of 0-1, baseline FAMCARE-P13 and global quality of life scores were high in both cohorts, indicating little room for improvement in the primary outcome. Implementation may also have been influenced by objective and subjective factors. We did not provide a summary recommendation or score indicating referral appropriateness, and oncologists’ frequency of PCRS review and perceived usefulness are unknown. These aspects warrant further research to optimize PCRS usability and clinical impact.

Nurse collection of PROMs requires additional resources and could be replaced by automated digital collection and reporting within the EMR. Further analyses of PCRS item and domain scores are needed to identify candidate referral thresholds and test them prospectively, potentially improving needs identification and clinical effectiveness.

The lower cumulative incidence of referral after PCRS implementation in biliary-pancreatic-gastric and head and neck cancers warrants cautious interpretation. These differences arose from an exploratory secondary analysis, were not statistically significant (aggregated poor-prognosis group: 24-week cumulative incidence 28% vs 20%, *P* = .184; sHR 0.68, 95% CI 0.31-1.51), and were based on small subgroups. They may reflect a tumor-specific ceiling effect, whereby referral in these cancers was strongly diagnosis-driven before implementation and may have been modified by PCRS use.

There are other limitations in our study methodology and results. Despite the inclusive eligibility criteria making the study population comparable to the real-life outpatient advanced cancer population, the monocentric nature of the study limits generalization of the results. The different balance of prognostic factors between the 2 cohorts, with a better ECOG-PS and a longer interval from advanced disease diagnosis in the post-PCRS group, may be due to an unintentional selection. Furthermore, the 6-month follow-up is limited for some outcomes, such as EoL care aggressiveness criteria, and should therefore be extended to collect more comprehensive data. In the context of the global shortage of healthcare professionals, future models of EPC integration should combine structured referral systems with artificial intelligence support to promote more sustainable, scalable, and personalized cancer care pathways. A significant number of eligible patients were not enrolled (Figure 1), but the reason for non-enrollment does not seem to suggest a selection bias. Enrollment began after the acute COVID-19 phases in Italy, with both cohorts recruited during progressively normalized hospital activity; residual effects on the Pre-PCRS cohort cannot be excluded, particularly caregiver access restrictions, reduced elective admissions, and increased telephone follow-up, which may have influenced healthcare utilization and caregiver satisfaction. Enrollment was limited by research staff availability, leaving a risk of selection bias. Diagnoses prompting hospitalization or ER attendance were not recorded, and the small number of referred patients precluded formal analysis of predictors of PC clinic attendance. Finally, although all comparisons of healthcare utilization were adjusted for baseline clinical confounders through IPTW, the propensity score model did not include factors specifically associated with the risk of hospitalization, such as comorbidity burden, previous admissions, or baseline symptom intensity, and residual confounding cannot be excluded.

## Conclusion

The implementation of the PCRS within the electronic medical record did not improve patient satisfaction with care or quality of life and did not increase the overall rate of palliative care referral, but was associated, in exploratory analyses, with changes in the timing and pattern of referral across tumor types. Whether these changes in referral reflect more appropriate, needs-based care, and whether they translate into lower healthcare utilization and less aggressive end-of-life care, should be tested in larger studies with a more pragmatic implementation strategy.

## Supplementary Material

oyag428_Supplementary_Data

## Data Availability

The data underlying this article will be shared on reasonable request to the corresponding author.

## References

[1] Haroen H , Maulana S, Harun H, et al The benefits of early palliative care on psychological well-being, functional status, and health-related quality of life among cancer patients and their caregivers: a systematic review and meta-analysis. BMC Palliat Care. 2025;24:120. 10.1186/s12904-025-01737-y PMC12036283 40296046

[2] Vanbutsele G , Van Belle S, Surmont V, et al The effect of early and systematic integration of palliative care in oncology on quality of life and health care use near the end of life: a randomised controlled trial. Eur J Cancer. 2020;124:186-193. 10.1016/j.ejca.2019.11.009 31812934

[3] Huo B , Song Y, Chang L, et al Effects of early palliative care on patients with incurable cancer: a meta-analysis and systematic review. Eur J Cancer Care (Engl). 2022;31:e13620. 10.1111/ecc.13620 35612356

[4] Conlon MSC , Caswell JM, Knight A, et al Impact of comprehensive hospice palliative care on end-of-life care: a propensity-score–matched retrospective observational study. CMAJ Open. 2019;7:E197-E202. 10.9778/cmajo.20180148 PMC6450796 30948648

[5] Dussel V , Orellana L, Holder R, et al A multisite randomized controlled trial of an early palliative care intervention in children with advanced cancer: the PediQUEST response study protocol. PLoS One. 2022;17:e0277212. 10.1371/journal.pone.0277212 PMC9642881 36346783

[6] Scarfò L , Karamanidou C, Doubek M, et al MyPal ADULT study protocol: a randomised clinical trial of the MyPal ePRO-based early palliative care system in adult patients with haematological malignancies. BMJ Open. 2021;11:e050256. 10.1136/bmjopen-2021-050256 PMC8565565 34728446

[7] Cloyd JM , Khatri R, Sarna A, et al Early palliative care following aborted cancer surgery: results of a prospective feasibility trial. Ann Surg Open. 2024;5:e520. 10.1097/AS9.0000000000000520 PMC11661761 39711683

[8] Nelson JE , Curtis JR, Mulkerin C, et al; Improving Palliative Care in the ICU (IPAL-ICU) Project Advisory Board. Choosing and using screening criteria for palliative care consultation in the ICU: a report from the improving palliative care in the ICU (IPAL-ICU) advisory board. Crit Care Med. 2013;41:2318-2327. 10.1097/CCM.0b013e31828cf12c 23939349

[9] King S , Sinnarajah A, Ahmed S, et al Patient-Rated acceptability of automatic palliative care referral: a prospective cohort study. J Pain Symptom Manage. 2025;69:465-472. 10.1016/j.jpainsymman.2025.01.021 39914578

[10] Ullrich A , Wilde S, Müller V, et al Acceptance and benefits of two different strategies to timely integrate specialist palliative care into routine cancer care: a randomized pilot study. Oncol Res Treat. 2022;45:118-129. 10.1159/000521077 34818660

[11] Fliedner M , Zambrano S, Schols JM, et al An early palliative care intervention can be confronting but reassuring: a qualitative study on the experiences of patients with advanced cancer. Palliat Med. 2019;33:783-792. 10.1177/0269216319847884 31068119

[12] Temel JS , Greer JA, Muzikansky A, et al Early palliative care for patients with metastatic non–small-cell lung cancer. N Engl J Med. 2010;363:733-742. 10.1056/NEJMoa1000678 20818875

[13] Ferrell BR , Temel JS, Temin S, et al Integration of palliative care into standard oncology care: American Society of Clinical Oncology Clinical Practice guideline update. J Clin Oncol. 2017;35:96-112. 10.1200/JCO.2016.70.1474 28034065

[14] Kaasa S , Loge JH, Aapro M, et al Integration of oncology and palliative care: a Lancet oncology commission. Lancet Oncol. 2018;19:e588–653-e653. 10.1016/S1470-2045(18)30415-7 30344075

[15] Guo L , LeBlanc TW, Smith CB, et al Early specialist palliative care in patients with metastatic cancer: a population-based cohort study. J Pain Symptom Manage. 2025;70:140-148.e2. 10.1016/j.jpainsymman.2025.04.005 PMC12229770 40252859

[16] Bergerot CD , Soto-Perez-de-Celis E, Thompson C, et al Navigating challenges in palliative care: a survey on ASCO guideline adherence among health care providers in low- and middle-income countries. JCO Glob Oncol. 2025 May; 11: e2400625. 10.1200/GO-24-00625 PMC12123599 40403199

[17] Sanders JJ , Temin S, Ghoshal A, et al Palliative care for patients with cancer: ASCO guideline update. J Clin Oncol. 2024;42:2336-2357. 10.1200/JCO.24.00542 38748941

[18] Gemmell R , Yousaf N, Droney J. “Triggers” for early palliative care referral in patients with cancer: a review of urgent unplanned admissions and outcomes. Support Care Cancer. 2020;28:3441-3449. 10.1007/s00520-019-05179-0 31792880

[19] Yang GM , Ke Y, Ng XH, et al Proactive symptom monitoring to initiate timely palliative care for patients with advanced cancer: a randomized controlled trial. Support Care Cancer. 2025;33:249. 10.1007/s00520-025-09311-1 40038129

[20] Zimmermann C , Pope A, Hannon B, et al Symptom screening with targeted early palliative care (STEP) versus usual care for patients with advanced cancer: a mixed methods study. Support Care Cancer. 2023;31:404. 10.1007/s00520-023-07870-9 37341839

[21] Rodin R , Swami N, Pope A, et al Impact of early palliative care according to baseline symptom severity: secondary analysis of a cluster-randomized controlled trial in patients with advanced cancer. Cancer Med. 2022;11:1869-1878. 10.1002/cam4.4565 PMC9041071 35142091

[22] Hui D , Mori M, Watanabe SM, et al Referral criteria for outpatient specialty palliative cancer care: an international consensus. Lancet Oncol. 2016;17:e552–9-e559. 10.1016/S1470-2045(16)30577-0 27924753

[23] Hui D , Meng YC, Bruera S, et al Referral criteria for outpatient palliative cancer care: a systematic review. Oncologist. 2016;21:895-901. 10.1634/theoncologist.2016-0006 PMC4943399 27185614

[24] Brunelli C , Zecca E, Pigni A, et al Outpatient palliative care referral system (PCRS) for patients with advanced cancer: an impact evaluation protocol. BMJ Open. 2022;12:e059410. 10.1136/bmjopen-2021-059410 PMC9621186 36307164

[25] Pigni A , Alfieri S, Caraceni AT, et al Development of the palliative care referral system: proposal of a tool for the referral of cancer patients to specialized palliative care. BMC Palliat Care. 2022;21:209. 10.1186/s12904-022-01094-0 PMC9816370 36443700

[26] Grassi L , Johansen C, Annunziata MA, et al Italian Society of Psycho-Oncology Distress Thermometer Study Group. Screening for distress in cancer patients: a multicenter, nationwide study in Italy. Cancer. 2013;119:1714-1721. 10.1002/cncr.27902 23423789

[27] Moro C , Brunelli C, Miccinesi G, et al Edmonton symptom assessment scale: Italian validation in two palliative care settings. Support Care Cancer. 2006;14:30-37. 10.1007/s00520-005-0834-3 15937688

[28] Groenvold M , Petersen MA, Aaronson NK, et al EORTC Quality of Life Group. The development of the EORTC QLQ-C15-PAL: a shortened questionnaire for cancer patients in palliative care. Eur J Cancer (1965). 2006;42:55-64. 10.1016/j.ejca.2005.06.022 16162404

[29] Massa G , Tinè G, Zecca E, et al The Italian version of the FAMCARE-P13 questionnaire: a validation study. J Pain Symptom Manage. 2025;70:392-399.e2. 10.1016/j.jpainsymman.2025.07.017 40738415

[30] Chattat R , Ottoboni G, Zeneli A, et al The Italian version of the FAMCARE scale: a validation study. Support Care Cancer. 2016;24:3821-3830. 10.1007/s00520-016-3187-1 27067593

[31] Mack JW , Weeks JC, Wright AA, et al End-of-life discussions, goal attainment, and distress at the end of life: predictors and outcomes of receipt of care consistent with preferences. J Clin Oncol. 2010;28:1203-1208. 10.1200/JCO.2009.25.4672 PMC2834470 20124172

[32] Austin PC , Stuart EA. Moving towards best practice when using inverse probability of treatment weighting (IPTW) using the propensity score to estimate causal treatment effects in observational studies. Stat Med. 2015;34:3661-3679. 10.1002/sim.6607 PMC4626409 26238958

[33] Rosenbaum PR , Rubin DB. The central role of the propensity score in observational studies for causal effects. Biometrika. 1983;70:41-55. 10.1093/biomet/70.1.41

[34] Aalen OO , Johansen S. An empirical transition matrix for non-homogeneous Markov chains based on censored observations. Scand J Stat. 1978;5:141-150. https://www.jstor.org/stable/4615704

[35] Conner SC , Trinquart L. Estimation and modeling of the restricted mean time lost in the presence of competing risks. Stat Med. 2021;40:2177-2196. 10.1002/sim.8896 PMC8889377 33567477

[36] Haun MW , Estel S, Rücker G, et al Early palliative care for adults with advanced cancer. Cochrane Database Syst Rev. 2017;6:CD011129. 10.1002/14651858.CD011129.pub2 PMC6481832 28603881

[37] Temel JS , Jackson VA, El-Jawahri A, et al Stepped palliative care for patients with advanced lung cancer: a randomized clinical trial. JAMA. 2024;332:471-481. 10.1001/jama.2024.10398 PMC11145511 38824442

[38] Caraceni A , Lo Dico S, Zecca E, et al Outpatient palliative care and thoracic medical oncology: referral criteria and clinical care pathways. Lung Cancer. 2020;139:13-17. 10.1016/j.lungcan.2019.10.003 31704278

