# Supplementary material for: A novel referral system to optimize timing to specialized palliative care in patients with advanced cancer: an interventional study
Source: Oncologist. 2026 Oct 28;31(12):oyag428. doi: 10.1093/oncolo/oyag428 (PMC13742279; doi:10.1093/oncolo/oyag428)
Supplement: Supplementary file 1 [file oyag428_supplementary_data.zip › Supplementary_Data/Supplementary Materials_clean_R2.docx]

**Supplementary Materials**

**List of Abbreviations**

ASCO: American Society of Clinical Oncology
ECOG PS: Eastern Cooperative Oncology Group Performance Status
EMR: Electronic Medical Record
EoL: End of Life
EORTC QLQ-C15-PAL: European Organisation for Research and Treatment of Cancer Quality of Life Questionnaire-Core 15 Palliative
EPC: Early Palliative Care
ER: Emergency Room
ESAS: Edmonton Symptom Assessment Scale
FAMCARE: Family Satisfaction with Advanced Cancer Care scale
FAMCARE-P13: 13-item Family Satisfaction with Advanced Cancer Care questionnaire
FPCR: First Palliative Care Referral
IPTW: Inverse Probability of Treatment Weighting
PC: Palliative Care
PCRS: Palliative Care Referral System
PROMs: Patient-Reported Outcome Measures
QoL: Quality of Life
TFPCR: Time to First Palliative Care Referral

| **Domain** | **Criteria** | **Measure/Tool** | **Cut off/ Scoring** | **Evaluator** | **Maximum Score** |
| --- | --- | --- | --- | --- | --- |
| **PROMs**  **(0-7,5)** | **Physical symptoms** | ESAS (11 items; no anxiety and depression) | 0.5 points each symptom≥5 | Nurse/case manager before visit | **5.5** |
|  | **Psychological symptoms** | ESAS (anxiety and depression only) | at least one ≥5 = 1 point | Nurse/case manager before visit | **1** |
|  | **Social Problems** | Items from Distress Termometre | at least two = 1 point | Nurse/case manager before visit | **1** |
|  |  |  |  |  | **7,5** |
| **ProPlus**  **(0-9)** | **Performance Status** | ECOG | 0 = 0 points | Physician during visit | **4** |
|  |  |  | 1 = 1 point |  |  |
|  |  |  | 2 = 2 points |  |  |
|  |  |  | 3 = 3 points |  |  |
|  |  |  | 4 = 4 points |  |  |
|  | **Prognosis** | Clinical evaluation | 0-3 moths = 2 points  3-12 months = 1 point  > 12 months = 0 points | Physician during visit | **2** |
|  | **Decision making/ Communication issue** | when the oncologist has to inform the patient about: change of therapy, discontinuation of active treatment | change of therapy =  1 point.  Discontinuation of active treatment =  2 point | Physician during visit | **2** |
|  | **Patient or caregiver request** | Patient or caregiver require access to palliative care | Yes = 1 point  No = 0 points | Team (before and/or during visit) | **1** |
|  |  |  |  |  | **9** |
| **ClinC**  **(0-8)** | **Serious Comorbidities** | Charlson comorbidity Items (but tumour) | None = 0 points  One or Two = 1 point  >Two = 2 points | Team (before and/or during visit) | **2** |
|  | **Other Relevant Conditions** | Clinical evaluation |  | Team (before and/or during visit) | **6** |
|  |  | Brain metastase | Yes = 1 point  No = 0 points |  | **1** |
|  |  | Bone metastases | Yes = 1 point  No = 0 points |  | **1** |
|  |  | Abnormal blood levels^a^ | Yes = 1 point  No = 0 points |  | **1** |
|  |  | Neoplastic effusion | Yes = 1 point  No = 0 points |  | **1** |
|  |  | Cognitive impairment/ delirium | Yes = 1 point  No = 0 points |  | **1** |
|  |  | Other syndrome or complication due to cancer | Yes = 1 point  No = 0 points |  | **1** |
|  |  |  |  |  | **8** |
| **Maximum total score** |  |  |  |  | **24,5** |

**Supplementary Table 1.** Structure and scoring system of the Palliative Care Referral System (PCRS) ^25^. Multidimensional domains, corresponding assessment tools, scoring rules, evaluator, and maximum achievable score (total 24.5 points) are shown.

List of abbreviations: ECOG= Eastern Cooperative Oncology Group performance status; ESAS= Edmonton Symptom Assessment Scale; PCRS= Palliative Care Referral System.

**Supplementary Table 2**: Cumulative incidences of first palliative care referral at 4, 8, 12, and 24 weeks stratified by arm. Crude Cumulative incidence (CCI) of FPCR at 4, 8, 12, and 24 weeks are reported for the Pre-PCRS and Post-PCRS arm. The CCI were adjusted via IPTW.

|  | **4w** | **8w** | **12w** | **24w** | **p-value** |
| --- | --- | --- | --- | --- | --- |
| **Arm** | **CCI (95% CI)** | **CCI (95% CI)** | **CCI (95% CI)** | **CCI (95% CI)** | 0.894 |
| Pre-PCRS | 7% (4%, 10%) | 11% (7%, 16%) | 13% (8%, 17%) | 20% (15%, 26%) |  |
| Post-PCRS | 5% (2%, 8%) | 9% (5%, 13%) | 13% (8%, 18%) | 21% (15%, 27%) |  |

List of abbreviations: FPCR =first palliative care referral; sHR=subdistribution Hazard Ratio; CI= Confidence Interval; w=weeks; IPTW= Inverse probability of treatment weighting.

**Supplementary Table 3**: Restricted Mean Time Loss of first palliative care referral for Pre-PCRS and Post-PCRS at 4, 8, 12 and 24 weeks. The estimates for RMTL were adjusted via IPTW.

|  | **RMTL** | | **Δ RMTL** | **p-value** |  |
| --- | --- | --- | --- | --- | --- |
|  | Pre-PCRS | Post-PCRS |  |  |  |
| **4w** | 0.2 | 0.1 | -0.1 | 0.194 |  |
| **8w** | 0.5 | 0.4 | -0.1 | 0.525 |  |
| **12w** | 1.0 | 0.9 | -0.1 | 0.726 |  |
| **24w** | 3.0 | 3.2 | 0.2 | 0.806 |  |

List of abbreviations: RMTL = Restricted Mean Time Loss; RMTL delta = Restricted Mean Time Loss Difference; w=weeks; IPTW= Inverse probability of treatment weighting; w=weeks

**Supplementary Table 4**: Cumulative incidences of first palliative care referral at 4, 8, 12, and 24 weeks stratified by tumor type and arm. CCI estimates were adjusted via IPTW.

| **Tumor Type** | **4w** | **8w** | **12w** | **24w** | **p-value** |
| --- | --- | --- | --- | --- | --- |
|  | **CCI (95% CI)** | **CCI (95% CI)** | **CCI (95% CI)** | **CCI (95% CI)** |  |
| **Lung** |  |  |  |  | 0.184 |
| Pre-PCRS (N=34) | 16% (3%, 29%) | 22% (7%, 36%) | 25% (9%, 40%) | 27% (12%, 43%) |  |
| Post-PCRS (N=27) | 8% (0%, 19%) | 8% (0%, 19%) | 12% (0%, 26%) | 25% (7%, 42%) |  |
| **Colorectal** |  |  |  |  | 0.540 |
| Pre-PCRS (N=8) | 9% (0%, 27%) | 23% (0%, 53%) | 22% (0%, 52%) | 21% (0%, 52%) |  |
| Post-PCRS (N=14) | 8% (0%, 25%) | 17% (0%, 38%) | 42% (14%, 70%) | 55% (27%, 83%) |  |
| **Biliary-Pancreatic-Gastric** | | | | | 0.050 |
| Pre-PCRS (N=19) | 26% (7%, 46%) | 32% (11%, 53%) | 32% (11%, 53%) | 47% (25%, 70%) |  |
| Post-PCRS (N=18) | 0% (0%, 0%) | 12% (0%, 28%) | 12% (0%, 28%) | 33% (9%, 56%) |  |
| **Head & Neck** | | | | | 0.208 |
| Pre-PCRS (N=33) | 6% (0%, 14%) | 10% (0%, 20%) | 10% (0%, 20%) | 17% (4%, 30%) |  |
| Post-PCRS (N=32) | 0% (0%, 0%) | 3% (0%, 8%) | 3% (0%, 8%) | 10% (0%, 21%) |  |
| **Sarcomas** | | | | | 0.852 |
| Pre-PCRS (N=30) | 0% (0%, 0%) | 2% (0%, 7%) | 5% (0%, 12%) | 17% (3%, 31%) |  |
| Post-PCRS (N=32) | 3% (0%, 9%) | 7% (0%, 16%) | 7% (0%, 16%) | 12% (1%, 23%) |  |
| **Breast** | | | | | 0.356 |
| Pre-PCRS (N=31) | 4% (0%, 11%) | 4% (0%, 11%) | 6% (0%, 14%) | 8% (0%, 17%) |  |
| Post-PCRS (N=30) | 4% (0%, 12%) | 11% (0%, 23%) | 14% (1%, 26%) | 21% (6%, 37%) |  |
| **Prostate** | | | | | 0.002 |
| Pre-PCRS (N=9) | 0% (0%, 0%) | 0% (0%, 0%) | 0% (0%, 0%) | 0% (0%, 0%) |  |
| Post-PCRS (N=9) | 24% (0%, 55%) | 39% (3%, 75%) | 67% (35%, 99%) | 70% (39%, 100%) |  |
| **Bladder & Kidney** | | | | | 0.630 |
| Pre-PCRS (N=22) | 0% (0%, 0%) | 5% (0%, 15%) | 5% (0%, 15%) | 19% (3%, 35%) |  |
| Post-PCRS (N=23) | 4% (0%, 13%) | 4% (0%, 13%) | 10% (0%, 23%) | 10% (0%, 23%) |  |

List of abbreviations: FPCR =first palliative care referral; CCI=Crude cumulative incidence; CI =Confidence Interval; w=weeks; IPTW= Inverse probability of treatment weighting.

**Supplementary Table 5**: Cumulative incidences of first palliative care referral at 4, 8, 12, and 24 weeks stratified by arm and aggregated tumor category. Tumor types were grouped according to similar prognosis. CCI estimates were adjusted via IPTW

|  | **4w** | **8w** | **12w** | **24w** | **p-value** |
| --- | --- | --- | --- | --- | --- |
|  | **CCI (95% CI)** | **CCI (95% CI)** | **CCI (95% CI)** | **CCI (95% CI)** |  |
| **Biliary-Thoracic/Other-B&K** | | | | | 0.184 |
| Pre-PCRS (N=45) | 8% (0%, 15%) | 14% (3%, 24%) | 14% (3%, 24%) | 28% (14%, 42%) |  |
| Post-PCRS (N=46) | 5% (0%, 11%) | 10% (1%, 19%) | 12% (2%, 23%) | 20% (8%, 32%) |  |
| **Sarcomas-Lung-H&N-Colorectal** | | | | | 0.540 |
| Pre-PCRS (N=105) | 9% (3%, 15%) | 13% (6%, 20%) | 15% (8%, 23%) | 21% (13%, 30%) |  |
| Post-PCRS (N=105) | 4% (0%, 9%) | 7% (2%, 12%) | 11% (5%, 17%) | 19% (12%, 27%) |  |
| **Breast-Prostate** | | | | | 0.050 |
| Pre-PCRS (N=40) | 3% (0%, 9%) | 3% (0%, 9%) | 5% (0%, 11%) | 6% (0%, 14%) |  |
| Post-PCRS (N=39) | 7% (0%, 16%) | 16% (4%, 28%) | 24% (10%, 37%) | 30% (16%, 45%) |  |

List of abbreviations: Biliary = Biliary pancreatic gastric; B&K = Bladder and Kidney; H&N = Head and Neck; CCI=Crude cumulative incidence; CI =Confidence Interval; w=weeks; IPTW= Inverse probability of treatment weighting.

**Supplementary Table 6**: Restricted Mean Time Loss of first palliative care referral stratified by aggregated tumour type and arm at 4, 8, 12 and 24 weeks. RMTL estimates were adjusted via IPTW.

|  | **RMTL** | | **Δ RMTL** | **p-value** |  |
| --- | --- | --- | --- | --- | --- |
|  | Pre-PCRS | Post-PCRS |  |  |  |
| **Biliary-Thoracic/Other-B&K** | | | | | |
| **4w** | 0.2 | 0.1 | -0.1 | 0.484 |  |
| **8w** | 0.6 | 0.3 | -0.3 | 0.452 |  |
| **12w** | 1.1 | 0.8 | -0.3 | 0.613 |  |
| **24w** | 3.9 | 2.8 | -1.1 | 0.465 |  |
| **Sarcomas-Lung-H&N-Colorectal** | | | | | |
| **4w** | 0.3 | 0.1 | -0.2 | 0.113 |  |
| **8w** | 0.6 | 0.3 | -0.3 | 0.224 |  |
| **12w** | 1.2 | 0.7 | -0.5 | 0.215 |  |
| **24w** | 3.4 | 2.8 | -0.6 | 0.517 |  |
| **Breast-Prostate** | | | | | |
| **4w** | - | - | - | - |  |
| **8w** | - | - | - | - |  |
| **12w** | 0.4 | 1.7 | 1.3 | 0.032 | * |
| **24w** | 1.2 | 5.2 | 4.1 | 0.008 | ** |

List of abbreviations: RMTL = Restricted Mean Time Loss; RMTL delta = Restricted Mean Time Loss Difference; Biliary = Biliary pancreatic gastric; B&K = Bladder and Kidney; H&N = Head and Neck; w=weeks.

**Supplementary Table 7**: Fine and Gray models for first palliative care referral, by aggregated tumor category.
sHRs are derived from an IPTW adjusted Fine and Gray models. Tumor types were aggregated according to similar prognosis

|  | **sHR** | **95% CI** | **p-value** |
| --- | --- | --- | --- |
| **BILIARY-THORACIC/OTHER-B&K** |  |  |  |
| **Arm** |  |  | 0.347 |
| Pre-PCRS | — | — |  |
| Post-PCRS | 0.68 | 0.31, 1.52 |  |
| **SARCOMAS-LUNG-H&N-COLORECTAL** |  |  |  |
| **Arm** |  |  | 0.560 |
| Pre-PCRS | — | — |  |
| Post-PCRS | 0.84 | 0.47, 1.50 |  |
| **BREAST-PROSTATE** |  |  |  |
| **Arm** |  |  | 0.065 |
| Pre-PCRS | — | — |  |
| Post-PCRS | 2.25 | 0.95, 5.34 |  |

List of abbreviations: Biliary = Biliary pancreatic gastric; B&K = Bladder and Kidney; H&N = Head and Neck; sHR=subdistribution Hazard Ratio; CI= Confidence Interval.

List of abbreviations: FPCR =first palliative care referral; Biliary = Biliary pancreatic gastric; B&K = Bladder and Kidney; H&N = Head and Neck. List of abbreviations: IPTW= Inverse probability of treatment weighting.

**Supplementary Figure 1. First palliative care referral cumulative incidence curves stratified by aggregated tumor category.**

IPTW adjusted FPCR cumulative incidence curves of Biliary-Thoracic/Other-B&K (panel A), Sarcomas-Lung-H&N-Colorectal (panel B), and Breast-Prostate (panel C) cancer are represented with blue and red line representing the cumulative incidence. Curves of Pre- and Post-PCRS respectively. Tumor types were aggregated by similar prognosis.

**Supplementary Figure 2.** Screenshot of the PCRS electronic form in Italian, with the 14 items shown in Supplementary Table 1. It has three sections: nurse-completed symptom and distress counts, oncologist-completed performance status, prognosis, communication and patient-request items, and team-completed comorbidity and clinical complication items with yes/no fields. No total referral recommendation is shown.

ECOG: Eastern Cooperative Oncology Group; ESAS-r: revised Edmonton Symptom Assessment System; PCRS: Palliative Care Referral System; PROMs: patient-reported outcome measures.
